# Complicated Liver Cystic Echinococcosis—A Comprehensive Literature Review and a Tale of Two Extreme Cases

**DOI:** 10.3390/tomography10060070

**Published:** 2024-06-11

**Authors:** Valentin Calu, Octavian Enciu, Elena-Adelina Toma, Radu Pârvuleţu, Dumitru Cătălin Pîrîianu, Adrian Miron

**Affiliations:** 1 Elias University Emergency Hospital, 011461 Bucharest, Romania; drcalu@yahoo.com (V.C.); adelina.toma@gmail.com (E.-A.T.); radu.parvuletu@gmail.com (R.P.); dramiron@yahoo.com (A.M.); 2 Department of Surgery, Carol Davila University of Medicine and Pharmacy, 020021 Bucharest, Romania

**Keywords:** liver hydatid cyst, peritoneal rupture, laparoscopic surgery, ERCP, biliary fistula

## Abstract

Cystic echinococcosis is a zoonotic parasitic disease that affects the liver in more than 70% of cases, and there is still an underestimated incidence in endemic areas. With a peculiar clinical presentation that ranges from paucisymptomatic illness to severe and possibly fatal complications, quality imaging and serological studies are required for diagnosis. The mainstay of treatment to date is surgery combined with antiparasitic agents. The surgical armamentarium consists of open and laparoscopic procedures for selected cases with growing confidence in parenchyma-sparing interventions. Endoscopic retrograde cholangiopancreatography (ERCP) is extremely useful for the diagnosis and treatment of biliary fistulas. Recent relevant studies in the literature are reviewed, and two complex cases are presented. The first patient underwent open surgery to treat 11 liver cysts, and during the follow-up, a right pulmonary cyst was diagnosed that was treated by minimally invasive surgery. The second case is represented by the peritoneal rupture of a giant liver cyst in a young woman who underwent laparoscopic surgery. Both patients developed biliary fistulas that were managed by ERCP. Both patients exhibited a non-specific clinical presentation and underwent several surgical procedures combined with antiparasitic agents, highlighting the necessity of customized treatment in order to decrease complications and successfully cure the disease.

## 1. Introduction

Cystic echinococcosis (CE) or hydatidosis is a zoonotic parasitic illness caused by the larval stages of Echinococcus granulosus. The hydatid cyst, which is the larval stage of Echinococcus, is a bladder-like cyst that develops in various organs and tissues in response to the creation of oncospheres by Echinococcus tape worms. The epidemiology and management of hydatidosis are often viewed as veterinary medicine concerns as the disease is normally managed by removing parasites from animals [1]. 

Echinococcus granulosus is widely encountered in extended endemic areas comprising countries of the temperate zones (Romania included), causing cystic hydatid disease which can at times be a life-threatening condition [2]. Due to the facility of long-distance travel and migration, the disease is present globally, and although it was once believed to be more restricted in geographic distribution, it is now considered an emerging disease [3].

Close collaboration between veterinarians and public health specialists in the prevention and control of illness is essential. Due to the close relationship between sheep and dogs, CE contributes to morbidity and even mortality in many regions of the world. Humans are most frequently the unfortunate intermediate hosts that become infected with Echinococcus. Intermediate hosts become infected by ingesting food or water contaminated with eggs from the feces of definitive hosts. The eggs release their larvae into the small bowel, which then traverse the intestinal wall to enter the liver via the portal system, where they grow into cysts [4].

Historically, the cyst consists of an outer fibrotic layer called the ectocyst or the pericyst and an inner fibrotic layer called the endocyst or the germinative membrane (internal membrane), from which brood capsules bearing protoscoleces (daughter cysts) disseminate into the cystic cavity. To be more exact, the pericyst is composed of compressed liver cells and fibrotic tissue, which is mostly a host reaction to the presence of the parasite and therefore does not belong to the parasite [5]. Therefore, only the internal membrane and the fluid it contains, as well as any live and infectious daughter cysts, should be the focus of any treatment.

The diagnosis of a simple CE is based on clinical suspicion; the most common symptom is discomfort in the right upper quadrant, and the most common findings are hepatomegaly or a palpable mass. Acute cholangitis is the most prevalent illness when hydatid cysts rupture in the biliary system. The most common signs of thoracic involvement are lower chest discomfort, a productive cough, and hemoptysis; bilioptysis is symptomatic of a bilio-bronchial fistula [6]. Pain in the right upper quadrant is the major clinical symptom (up to 88% of cases), followed by a high temperature (>39 °C) in up to 78% of cases, while elevated C-reactive protein (CRP) acts as a predictor of a complex hydatid disease [4].

Imaging is the standard method for detecting CE [7]. Ultrasonography (US) is essential for the study of localized abdominal CE. US can detect cysts in other organs, including the lung, if they are peripherally situated [8]. The staging of cysts is based on US features (Table 1—Gharbi and WHO classifications).

Magnetic resonance imaging (MRI) and computed tomography (CT) are supplementary imaging modalities for hepatic CE lesions. Typically, these techniques are employed for a preoperative assessment or in the event of complications. Ultrasound and magnetic resonance imaging are the imaging modalities that should be performed for pregnant patients to diagnose hydatid cysts [9]. 

The majority of patients with a primary infection have only one cyst; however, up to 20–40% of infected patients have several cysts [10]. The sizes and volumes of the cysts are the major determinants of patients’ symptoms, as well as the mass effect inside the organ and on surrounding tissues [11]. Nevertheless, a definitive diagnosis requires a combination of imaging and immunological studies [12].

Most patients are frequently asymptomatic for a long period of time after being infected with Echinococcus granulosus [13]. Clinical manifestations may be related to a toxic reaction due to the presence of the parasite or the local and mechanical effects depending on the location and nature of the cysts and the presence of complications [14]. Even though the presence of the cyst is only correlated with symptoms at a late stage of infection, almost all immunodiagnostic techniques (for detecting both antibodies and/or antigens) are still mainly used for laboratory research [15]. The field application of these techniques should be undertaken in endemic areas in order to take proper public health measures for eradication [16].

The synergistic action of surgery (either conservative or aggressive), PAIR (puncture–aspiration–injection–reaspiration), antiparasitic medicines, and active surveillance in some situations represent the treatment for this condition [17,18,19]. 

Historically, the standard treatment for liver CE has been surgery followed by medical treatment with antiparasitic agents [20]. Progress has been made over decades, from aggressive to conservative resections and from open to minimally invasive surgery with fewer complications.

PAIR therapy under ultrasound or CT guidance is indicated for type I–III cysts that are smaller than 5 cm without biliary communication [21]. PAIR is not an upfront indication, but it should be offered to patients who are poor candidates for surgery and to patients refusing surgery, and it must be associated with oral Albendazole.

According to the American College of Gastroenterology Guidelines, severe hydatid cysts with many vesicles, daughter cysts, fistulas, rupture, bleeding, or secondary infection are candidates for open or laparoscopic surgery based on available expertise [22]. 

Despite advancements in both hydatid disease detection and treatment procedures, recurrence remains a serious issue in the management of this disease, with an average re-occurrence period of over 10 years [23]. The liver remains the most common site for hydatic cysts (in about 50–70% of cases), followed by the lung, spleen, kidney, bones, brain, and other uncommon localizations [24,25]. In 0.16–16% of cases, hepatic hydatid disease may involve the diaphragm or thoracic cavity [26].

## 2. Materials and Methods

This is a comprehensive narrative review driven by the complex management of the presented cases. The literature research included Pubmed and Google Scholar with the terms “complicated liver hydatid cyst”, “biliary fistula”, “ERCP”, and “peritoneal rupture”, with the main focus on comparative case management and the most recent published guidelines. Relevant and relatively recent publications were used for data extraction with emphasis on diagnosis, surgical treatment, and the management of rare complications. Published papers from both endemic and non-endemic areas that treat patients who migrated from endemic areas were used.

## 3. Case Reports

### 3.1. Case 1

The first case concerns an 18-year-old female living in a rural area with no relevant surgical or medical history, who was admitted in our department at Elias University Emergency Hospital for widespread abdominal discomfort and significant distension. 

The clinical examination revealed an abdominal mass extending from the epigastric area to the right hypochondrium, in addition to diffuse abdominal discomfort. Plasma fibrinogen levels of 664 mg/dL (normal range: 200–400 mg/dL) were indicative of non-specific inflammatory illness. Otherwise, leucocytes, granulocytes, liver enzymes, bilirubin, and lipase were all within normal ranges. 

**Figure 1:**
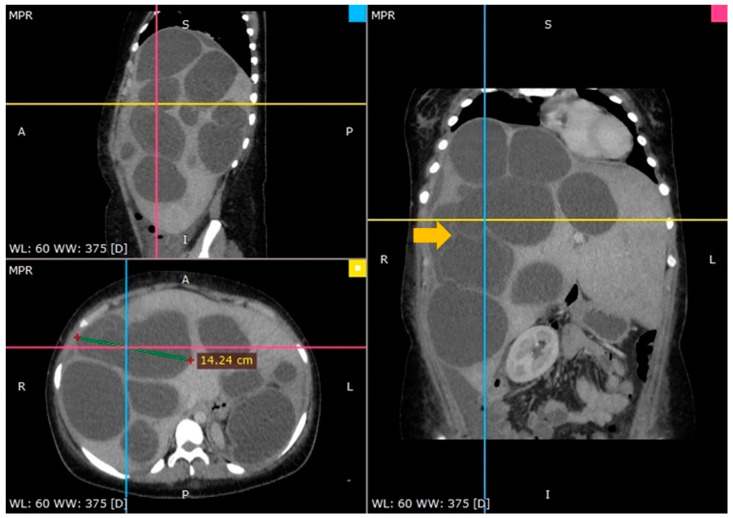
A CT scan showing multiple hydatid cysts in an enormously enlarged liver (arrow—floating internal membrane).

An ultrasound of the abdomen indicated a huge abdominal mass with several cysts spreading from the right hypochondrium to the pelvis. A computed tomography (CT) scan confirmed the diagnosis and offered a thorough image of the lesion: several enormous hydatid cysts that spanned the whole right and left lobes of the liver. The largest cyst measured almost 15 cm, and one cyst had the germinal layer detached (Figure 1).

In accordance with the parasitologist’s recommendations, the patient was administered Albendazole for 8 weeks (400 mg twice daily) prior to surgery after serological testing (IgG ELISA) confirmed the echinococcal infection.

**Figure 2:**
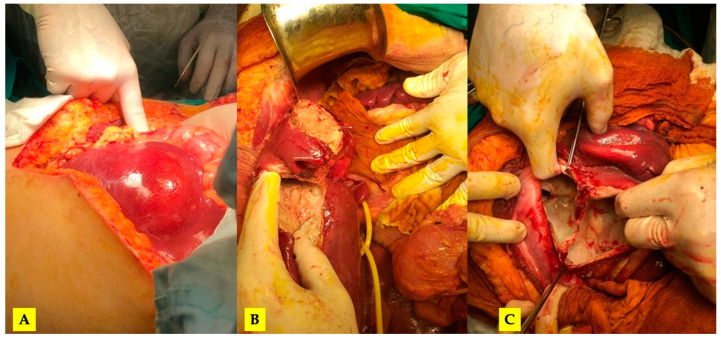
(**A**) The initial intraoperative aspect; (**B**) mobilized liver, a pericystectomy of the most anterior cysts and Huang’s loop prepared for Pringle maneuver; (**C**) the inner aspect of the largest cyst (no bile leaks).

After completing the medical treatment, the patient was re-admitted for surgery. The severity of the lesions required an open surgical approach. Through the so-called “Mercedes star” incision, a complete mobilization of the liver was achieved, and Huang’s loop was prepared for the Pringle maneuver (Figure 2).

Eleven cysts, as described by intraoperative ultrasonography, were inactivated with betadine solution, their contents were removed, and a cystectomy with partial parenchyma-sparing peri-cystectomy was performed. A cholecystectomy was performed with intraoperative cholangiography, and transcystic biliary external drainage for adequate decompression was deemed necessary. Throughout the whole surgical procedure, the resulting hepatic cavities were thoroughly examined without the exposure of biliary contents.

A final abdominal ultrasound was performed to confirm complete resection. The remaining cavities were drained, and omentoplasty was performed for the caudal and larger cysts.

On the third postoperative day (POD3), a bile leak with a low output (under 300 mL/day) became evident via the tube draining the largest cavity. Endoscopic sphincterotomy was employed to decompress the common bile duct by ERCP, followed by a reduction in output. The transcystic drainage was extracted after two weeks, and the leak appeared sealed after four weeks. When the drainage flow rate was less than 10–15 mL/24 h, the remaining tubes were eventually removed in succession, with a US examination preceding each removal.

Three weeks after surgery, a right pleural effusion was identified and failed to respond to numerous ultrasound-guided thoracentesis procedures; thus, the placement of a chest tube was considered mandatory. The chest drain was removed after 5 more days, and the patient was discharged from the hospital after 53 days.

At the three-month follow-up, the CT scan revealed no evidence of recurrence, with fluid filling the biggest remnant cavity, the blood tests were normal, and the patient was in sound clinical condition. 

**Figure 3:**
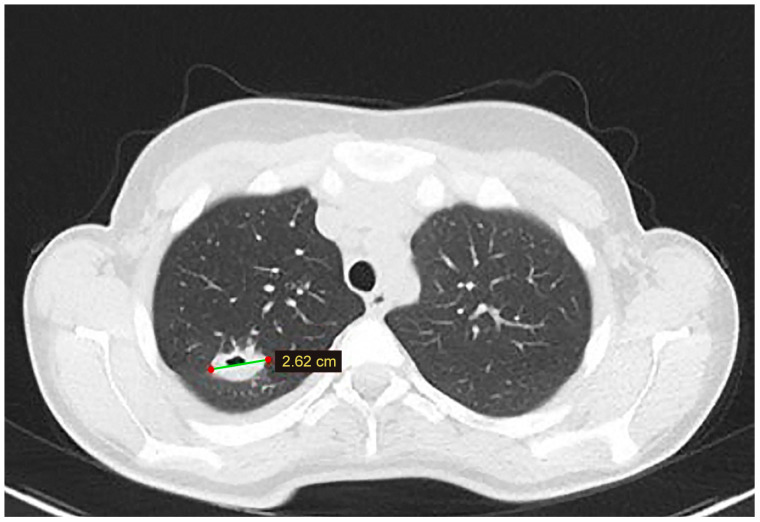
A lung CT scan at the 6-month follow-up—a cystic lesion is discovered in the superior lobe of the right lung.

At the six-month follow-up, the chest CT scan identified a cystic lesion in the upper lobe of the right lung. Even though the lesion was asymptomatic, given the prior history of extended liver disease and adequate Albendazole treatment, surgical removal was decided (Figure 3). The patient underwent video-assisted thoracoscopic surgery (VATS), and cystectomy was performed. The postoperative course was uneventful, and the patient was discharged four days after surgery.

The patient received Albendazole for eleven months after the abdominal intervention and remained under strict parasitological observation.

The CT scan performed after three years did not reveal any recurrence apart from one fluid-filled remnant cavity of 43/33 mm positioned at the level of segment 8. The liver shape was normal. 

### 3.2. Case 2

The second case is a 19-year-old female, also coming from a rural area, without any significant surgical or medical history, who presented to the emergency room with diffuse abdominal pain that had appeared 3 months prior but intensified in the last 2 days. 

A complete blood panel showed slight leukocytosis (12.30 × 10^3^/µL; normal range: 4.0–10.0 × 10^3^/µL) and mild jaundice (total bilirubin of 3.28 mg/dL; normal range: 0.2–1.2 mg/dL). 

**Figure 4:**
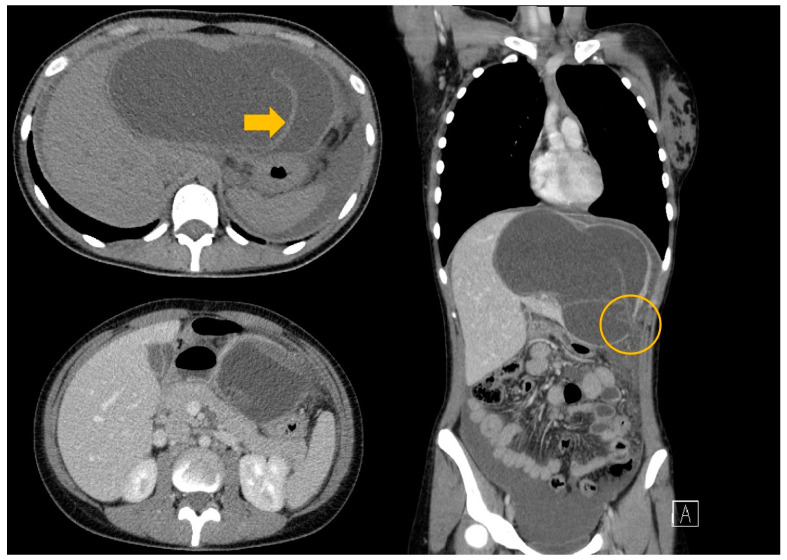
A CT scan of the thorax abdomen and pelvis with contrast medium—a giant liver hydatid cyst with rupture at the level of segment III with free peritoneal fluid (arrow—the floating internal membrane; circle—the rupture of the cyst).

An abdominal US revealed hepatomegaly and the presence of a large liver cyst with free fluid around the liver. Thus, suspicion of a ruptured hepatic hydatid cyst was raised. The CT scan diagnosed a hydatid cyst with detached germinal layer, a rupture at the level of the left hepatic lobe, and free fluid around the liver and in the recesses of the peritoneal cavity (Figure 4).

**Figure 5:**
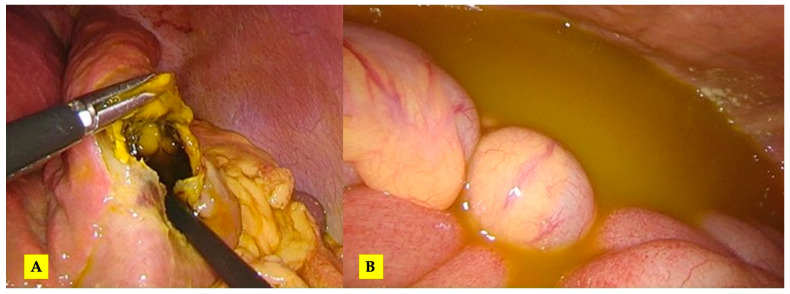
The intraoperative aspect: (**A**) evident rupture of the hydatid cyst at the level of segment III and (**B**) free peritoneal fluid in the peritoneal cavity.

The patient underwent emergency exploratory laparoscopy. An intraoperative examination revealed the presence of abundant peritoneal fluid with bile and cystic content from a ruptured liver hydatid cyst at the level of segments III and IV of the liver without any evident macroscopic bile duct leakage (underwater check of the cavity) (Figure 5). Having considered the age of the patient and the amount of liver parenchyma to be lost if resection was performed, partial cystectomy was deemed feasible. Abundant peritoneal lavage was performed with the drainage of the peritoneal cavity and of the remnant cavity. The diagnosis of hydatid disease was confirmed during index admission by specific tests (ELISA IgG = 7.023 index, index IgG > 1.100 positive). 

On POD 4, low-output (<300 mL/day) bile leak was diagnosed, but the patient did not exhibit any additional symptoms, so a watch-and-wait management method was decided. 

On POD 8, a laparoscopic exploration was performed due to important postoperative bleeding that became evident through the cavitary drainage tubes. The source of the bleeding was a small vessel in the pericyst corresponding to liver segment III and was controlled by diathermy. During exploration, the bile leak was not visualized, probably due to the size of the cyst, blood clots, and, most likely, a more medial location. 

**Figure 6:**
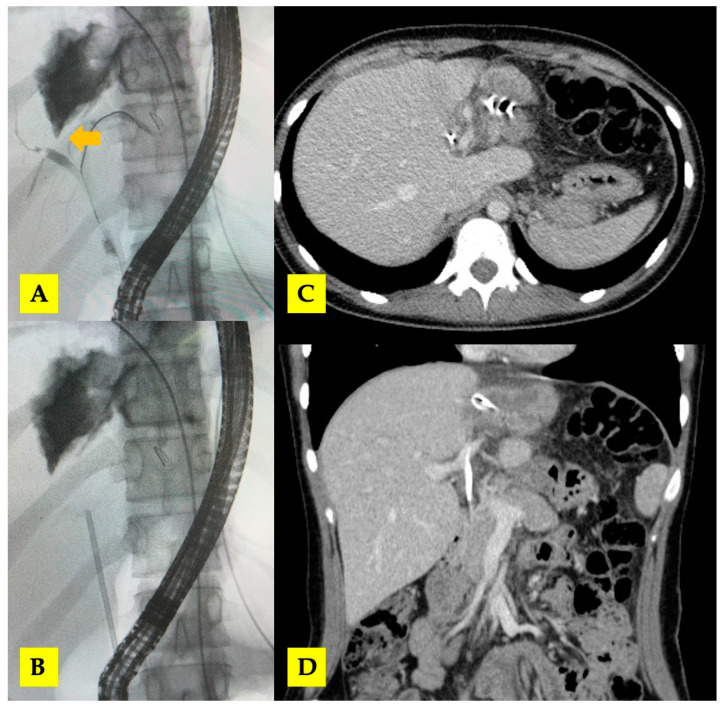
(**A**,**B**) ERCP with biliary stent placement (arrow—central biliary communication with right hepatic duct); (**C**,**D**) post-ERCP CT scan with biliary stent in situ.

By POD 10, the bile leak output increased to 350–400 mL/day and remained steady for the next 3 days, with the patient in stable clinical condition. ERCP was performed in POD 14, which demonstrated a significant biliary fistula with the right hepatic duct. A biliary (8Fr) stent was placed to improve biliary drainage. Subsequently, the output decreased to 200 mL/day, and the patient was discharged on POD 21 with both intrahepatic drainage tubes in situ (Figure 6). The drainage tubes were removed during the next 2 months after consecutive follow-up CT scans.

After discharge, the patient underwent antiparasitic treatment with Albendazole for 24 weeks (15 mg per kg of body weight per day divided into two doses, since the patient weighed 48 kg) after surgery and remained disease-free at the 2-year follow-up. The liver parenchyma expanded in a consistent manner, as expected.

## 4. Discussion

There are worrying reports from several endemic regions of the globe that echinococcosis-related hazards to human health are on the rise even after deworming programs [27,28].

The natural history of human hepatic hydatidosis is poorly described. Several ultrasonographic morphological characteristics indicate that the parasite undergoes its own evolution in the human liver from birth to death. Findings such as a completely calcified (dormant or dead) cyst in asymptomatic patients suggest that not all hydatid cysts require therapy. Yet, investigations have failed to establish a correlation between the presence of symptoms and the course of the disease [5].

The rationale for the elective treatment of a liver hydatid cyst is that it may enlarge and cause symptoms and complications, such as infection, jaundice or cholangitis from rupture into a biliary duct, and anaphylaxis.

While the majority of liver hydatid cysts are asymptomatic, some patients present with non-specific abdominal discomfort or tenderness in the epigastric area or the right upper abdomen quadrant. Typical clinical examination findings include an enlarged liver and, in rare instances, a palpable abdominal mass.

The non-specific clinical presentation (diffuse abdominal pain) of the two cases, as well as the slight biological changes, shows that it is possible that in young patients without comorbidities, the symptoms do not correlate, at least in an acute phase, with the degree or progression of the cysts and the severity of the disease. On the other hand, cystic rupture is almost always a terrible event with a high clinical impact.

The cysts that are the most susceptible to rupture in the peritoneal cavity are large, superficial, thin-walled hepatic hydatid cysts situated along the inferior or anterior surface of the liver. Rupture may occur spontaneously or following a small or harsh abdominal injury. Clinical manifestations may include anaphylactic shock, a severe abdominal condition, or both. A decreased cyst size, a defect in the cystic wall, detached membranes, and a change in the hydatid cyst’s architecture during follow-up are CT indicators of hepatic hydatid cyst rupture. This complication represents a significant threat to a patient’s life since it may result in anaphylactic shock or subsequent bacterial peritonitis. A CT scan may reveal imaging symptoms and findings of hepatic hydatid cyst rupture accompanied with ascites, intra-abdominal fat standing, and peritoneal enhancement. Early, correct diagnosis, and prompt and appropriate treatment are essential for avoiding negative effects.

The annual death rate due to echinococcosis is 0.007 per 10,000 individuals, with a fatality rate of 1.29 per 100 cases [29]. It has been estimated that as many as 60 percent of individuals identified and treated for hepatic hydatid disease have complex hydatid cysts (HCs) [30]. In two retrospective investigations, age (>40 years), cyst size (>10 cm), the number of cysts (>3), difficult HC, and conservative surgery were identified as high-risk variables in patients with HCs since they significantly increase morbidity and death rates [31]. Patients aged 70–79 had the highest reported death rates; echinococcosis is also a cause of death in infants who have not been operated on. Direct causes of death include the rupture of the parasite cyst and hepatic failure [29]. The rupture of a hydatid cyst results in an allergic reaction in 50 to 90% of cases due to the presence of antigenic cyst material in the surrounding tissues.

Patients usually seek medical assistance with longstanding disease, when complications have typically already occurred. This is the situation in the vast majority of cases and the reason why ultrasound screening in endemic areas has been advocated [4].

Individuals living in rural areas of endemic regions and working in high-risk occupations may be subjected to frequent (annual) screening. Likewise, individuals living in the same household with infected patients that undergo close follow-ups after treatment should be screened. Even though it is operator-dependent, abdominal ultrasound remains the gold standard imaging method for screening. At an average growth rate of 1–20 mm per year for hepatic hydatid cysts, serological tests and biomarkers are required in a high-risk population [32]. In certain cases with very unlikely localizations, a diagnosis might be obtained by fine needle aspiration and cytology [33,34]. Given the options for screening and advanced immunology studies published, one should keep in mind that the endemic areas for CE are low- to middle-income countries with limited budgets.

Besides serum immunoglobulin detected by ELISA, new antigens such as the protein membrane EG-06283, antigen 5, and antigen B are emerging as potential markers for the disease. Rapid diagnosis in endemic countries may improve control with medication and lower the need for surgical treatment [35,36]. The sensitivity and specificity for the diagnosis of EG-06283 are 92.59% and 84.62%, respectively, and while using antigen B, antigen 5, and IgG, sensitivity reaches 97%, and specificity reaches 95.7%.

Imaging, serological, and immunological procedures need to be performed together in order to arrive at a conclusive diagnosis. As far as radiological diagnosis is concerned, the basis for the diagnosis of hepatic hydatid cystic disease is an ultrasound examination due to its already-known benefits, which include the fact that it is quick, relatively easy to perform, inexpensive, and does not expose the patient to radiation. Nonetheless, a CT scan is still the most helpful method to establish problems and develop an appropriate treatment plan, as we achieved for both of these patients. The differential diagnosis of liver CE includes simple liver cysts, solitary bile duct cysts, hepatobiliary cystadenoma, hemangioma, and abscess [37,38].

Presently, efforts are being made to establish a standardization for treatment worldwide, and four distinct CE treatment regimen strategies based on the WHO cyst stage are recommended [39]. The first standardized classification of CE was presented by the WHO-IWGE back in 1995 [40]. As of 2009, the WHO-IWGE issued new guidelines for the diagnosis, treatment, and management of CE and AE. These guidelines were based on a consensus. Table 2 contains the most recent version of the pathognomonic characteristics of CE cysts in the United States, which was revised by the WHO. On the basis of the cyst’s size, form, and location, this classification is able to differentiate between cysts that are active, transitionally active, and dormant [41,42,43]. This is a step-wise approach for the treatment of CE, but it is worth mentioning that the surgical procedures required for treatment cannot be standardized. Even if for non-complicated disease, there is no consensus regarding the best type of intervention, for complicated disease (rupture and biliary fistula), the options for treatment are manifold, and most frequently, all options can and must be employed as in our presented cases.

The inactivation of the cyst, the prevention of intraoperative spillage of cystic contents, the elimination of all viable elements of the cyst, and the treatment of the residual cystic cavity through drainage and/or omentoplasty in order to prevent abscess formation are the goals of surgical treatment. The spectrum of the surgical operation ranges from an aggressive approach (pericystectomy or liver resection) all the way to a more cautious approach (the drainage or obliteration of the cavity or both) [44].

These surgical aims can be accomplished by either traditional open surgery or laparoscopic surgery [14]. Our first case could not be managed safely by minimally invasive surgery due to the extent of the disease. Since multiple cysts were present, we considered that intraoperative ultrasound was a very useful tool after the completion of the procedure to check for undiscovered remnant cysts that could possibly lead to early recurrence [45]. Also, if a liver resection is planned, major vascular vicinity must be revealed, and the hepatic anatomy should be clear to the surgeon beforehand. Depending on the experience of the surgical team, the laparoscopic approach can be used when the objectives of surgery can be safely achieved and there are no contraindications for anesthetizing patients for longer periods of time, as we performed in our second case. However, it must be assessed whether the benefit of a faster postoperative recovery corresponds to the effective eradication of the disease; thus, patient selection is paramount [46].

The use of endoscopic retrograde cholangiopancreatography (ERCP) as a diagnostic and therapeutic tool for the treatment of cystobiliary communications in patients with hepatic hydatid cyst disease is considered to be the gold standard [47]. Postoperatively, it is frequently necessary to use ERCP for the purpose of both detecting and treating bile leaks. Preoperative ERCP is needed for patients with jaundice, and it has been advocated for the prevention of intraoperative and postoperative biliary leaks in cases with liver hydatid cysts greater than 7.5 cm in diameter [48,49]. Routine ERCP for uncomplicated cysts cannot be recommended [49]. Compression in the adjacent parenchyma might obscure small biliary fistulas that become evident postoperatively. After surgery, bile leakage was observed in both of our patients, but it was effectively treated with ERCP. In the first case, sphincterotomy was sufficient, but in the second case, due to the high output and central location, sphincterotomy and biliary stent placement was considered mandatory. Although the cyst mainly affected the left liver, biliary communication affected the right hepatic duct. It has been reported that cystobiliary communications most frequently involve the right hepatic duct, while the left hepatic duct and the biliary confluence are less frequently involved [26].

Both patients received Albendazole; in the first case, the patient received Albendazole both before and after surgery, and in the second case, the patient only received it after surgery since the intervention was urgent, and follow-up CT scans revealed no evidence of hepatic recurrence in either patient. Although non-surgical treatment had poorer outcomes in the past, it is currently being revisited on account of new generations of anti-parasitic agents (such as Mebendazole and Praziquantel), and the association of Praziquantel with Albendazole seems to have a synergetic effect [50]. These anti-parasitic agents reduce ATP production, as well as cell division, egg production, and development; as a result, the parasite is immobilized and eventually dies in between 50 and 80 percent of cases after only three months of treatment, and some authors reported a sterilization rate of 95% [51].

In complicated liver disease, long standing illness is to be expected, and a follow-up is necessary both for recurrence and new lesions in other organs. Due to rapid growth, giant pulmonary lesions often occur in younger patients and are more symptomatic at presentation. In the first presented case, the pulmonary lesion was diagnosed at 6 months of follow-up after the liver intervention and a full course of Albendazole; thus, surgical intervention was deemed necessary [52]. The intervention was performed through minimally invasive surgery, and pulmonary resection was not necessary [53]. 

## 5. Conclusions

Even in cases of complicated liver hydatid cysts, the clinical presentation may be quite non-specific, and expeditious imaging may precede emergency surgery. Moreover, to achieve a cured status and/or reduce recurrence, multiple interventions may be required in addition to multidisciplinary management. This is especially true for the presented cases, one of which was represented by peritoneal rupture. A thorough long-term follow-up is needed for such cases, mainly because another location might be brewing in long-standing CE, as exemplified by the first presented case. Nonetheless, a cure can be achieved even in complicated cases employing minimally invasive techniques (laparoscopy, ERCP, and intraoperative ultrasonography) and conservative surgery.

## Figures and Tables

**Table 1:** Ultrasonographic WHO classification of hydatid cysts.

Gharbi Type	WHO Type	Cyst Morphology
**I**	**CE 1**	Unilocular anechoic lesion with “double line” sign
**II**	**CE 2**	Multiseptated rosette-like honeycomb cyst
**III**	**CE 3A**	Cyst with detached membranes (“water-lily” sign)
**III**	**CE 3B**	Cyst with daughter cysts in sold matrix
**IV**	**CE 4**	Cyst with heterogenous hypoechoic/hyperechoic contents No daughter cysts
**V**	**CE 5**	Solid plus calcified wall

**Table 2:** Treatment regimens recommended according to WHO type.

WHO Type	Main Characteristics	Viability	Treatment
**Cystic Echinococcosis 1**	Uniloculated cyst with “double wall” sign	Active cyst (viable)	ABZ 3-6 months (small cyst < 5 cm) **or** percutaneous treatment (PAIR) and 1-6 months ABZ (cysts 5–10 cm) **or** surgery + 1–3 months ABZ (cysts > 10 cm)
**Cystic Echinococcosis 2**	Uniloculated cyst with regular, vascular “septations” resembling daughter cysts	Active cyst (viable)	Surgery + 1–3 months ABZ **or** modified percutaneous treatment + 1–3 months ABZ **or** ABZ alone 3–6 months if cyst is small
**Cystic Echinococcosis 3A**	Continuous thin and regular membranes floating in cyst, resembling detached parasite layers	Transitional (variable viability)	ABZ 3-6 months (small cysts < 5 cm) **or** percutaneous treatment (PAIR) + 1–6 months ABZ (cysts 5–10 cm) **or** surgery + 1–3 months ABZ (cysts > 10 cm)
**Cystic Echinococcosis 3B**	Predominantly solid with daughter cysts	Transitional (variable viability)	Surgery + 1–3 months ABZ **or** modified percutaneous treatment + 1–3 months ABZ **or** ABZ 1–6 months if cyst is small
**Cystic Echinococcosis 4**	Parasite membranes embedded in heterogenous, avascular solid content (“ball of wool” appearance)	Inactive (low or no viability)	Watch and wait
**Cystic Echinococcosis 5**	Cysts with solid content with eggshell wall calcifications	Inactive (no viability)	Watch and wait

## Data Availability

The dataset is available upon request from the authors since further imaging data (CT/ERCP and intraoperative photos) can be offered upon request.
